# Research on individualized drug sensitivity detection technology based on bio-3D printing technology for precision treatment of gastrointestinal stromal tumors

**DOI:** 10.1515/biol-2025-1108

**Published:** 2025-05-27

**Authors:** Yang-Yang Tu, Zhong-Ting Huang, Zhi-Yong Zhang, Dong-Liang Liu, Hai-Long Qian

**Affiliations:** Department of Gastrointestinal Surgery, Ningbo Medical Center Li Hui Li Hospital, Ningbo, Zhejiang, 315000, China

**Keywords:** bio-3D printing technology, drug sensitivity testing, gastrointestinal stromal tumor

## Abstract

This study aims to analyze and explore whether tumor biological three-dimensional printing (3DP) models can serve as reliable preclinical model research tools and assist in the personalized treatment of gastrointestinal stromal tumor (GIST) patients. Ten GIST cases admitted to our hospital from May 2024 to September 2024 were included in the personalized treatment group. Patient-derived GIST 3DP models were established, and treatment plans were selected based on the results of drug sensitivity tests. The progression-free survival (PFS) of the personalized treatment group was compared with that of GIST patients who had progressed after first-line treatment and were admitted to our hospital before the study. Treatment safety was also assessed. Immunofluorescence staining technology was used to observe tumor markers in the 3DP tumor models and their corresponding parent tumor tissues, revealing a high degree of consistency, which indicates that the 3DP tumor models highly retain the histological characteristics of the parent tumor tissues. The median PFS of patients in the personalized treatment group was 6.1 months, compared to 5.3 months in the previous treatment group, with a statistically significant difference (*P*-value <0.05). The individualized drug sensitivity detection technology based on bio-3D printing technology, used for the personalized treatment of GIST patients who have progressed after first-line treatment, can benefit patients.

Gastrointestinal stromal tumors (GISTs) are the most common malignant mesenchymal tumors of the gastrointestinal tract. According to the National Cancer Institute in the United States, the incidence rate of GIST is approximately 1.5 per 100,000 people per year. Globally, GIST accounts for 1–2% of all gastrointestinal malignancies. In Europe, the incidence of GIST is estimated to be around 10 to 15 cases per million people per year [1]. In recent years, as our understanding of GIST has improved, diagnostic and therapeutic approaches for GIST have also been continuously refined.

GIST is highly resistant to conventional chemotherapy and radiotherapy. As human understanding of GIST has improved, tumor treatment is gradually shifting from standard protocols to precision medicine. Currently, in clinical practice, individualized and precise treatment plans can significantly improve patient prognosis. Compared to tumors such as gastric cancer and colorectal cancer, which involve multiple gene and pathway mutations, the mutation targets of GIST are relatively singular, mainly focusing on mutations in tyrosine protein kinase (c-KIT) (80%) and platelet-derived growth factor receptor alpha (PDGFRA) (10%), with the rest classified as wild-type [2]. Since the application of the tyrosine kinase inhibitor imatinib in the treatment of GIST, targeted therapy for GIST has achieved great success. For the first-line treatment of newly diagnosed GIST, a definite correlation has been established between the efficacy of imatinib and the primary gene mutations of GIST [3,4]. KIT mutations are mainly located in exon 11, with some mutations also present in exon 9. It has been clarified that for patients with KIT exon 11 mutations, imatinib can achieve an efficacy of over 90%; however, for patients with KIT exon 9 mutations, the treatment with imatinib needs to be intensified, and the prognosis is relatively worse compared to those with KIT exon 11 mutations. Additionally, patients with KIT exon 11 deletions and insertions have a worse prognosis than those with simple point mutations [5]. PDGFRA exon 18 mutations are typically D842V point mutations, and patients with this mutation are primarily resistant to imatinib, but avapritinib can achieve an efficacy close to 100% [6]. For wild-type patients without the aforementioned mutations, there are currently no clearly effective drugs in the first-line treatment.

With the rapid development of 3D bioprinting technology in recent years, 3D-bioprinted organ models have a broad prospect in the medical field. Currently, 3D-bioprinted tumor models have been widely applied in various solid tumors, including hepatocellular carcinoma, pancreatic cancer, colorectal cancer, ovarian cancer, and neuroblastoma. In a study by Sun et al., it was confirmed through genomic and histological analyses that colorectal cancer/colorectal cancer liver metastasis (CRC/CRLM) can effectively retain the parental tumor biomarkers and mutation spectra in 3D-bioprinted (3DP) tumor models. There is a significant correlation between the drug response in the CRLM biological three-dimensional printing (3DP) model and the clinical results of neoadjuvant chemotherapy. These findings suggest that patient-derived 3DP cancer models have great potential for the precise chemotherapy prediction and preclinical research in CRC/CRLM [7]. In terms of drug discovery, integrating intelligent cell culture systems and biosensors into 3D-bioprinted models can provide highly detailed and functional organ models for drug screening. By addressing current challenges in vascularization, electrophysiological control, and scalability, researchers can obtain more reliable and accurate drug development data, thereby reducing the risk of drug failure during clinical trials [8].

In patients with advanced GIST, most will develop secondary resistance to imatinib after approximately 2 years of first-line treatment. Currently, imatinib is the standard first-line treatment for GIST patients. However, once patients develop resistance to imatinib, the efficacy of second-line drugs available in the clinic is significantly lower than that of first-line treatment. In a previous retrospective study of 52 GIST patients who had progressed after first-line treatment at our hospital, the progression-free survival (PFS) time was only 4–6 months. Therefore, in the current treatment landscape for GIST, optimizing second-line treatment strategies and selecting sensitive second-line drugs as early as possible based on individualized drug sensitivity testing results are of paramount importance for achieving better prognosis. From an application perspective, 3D bioprinting technology holds higher clinical value, offering more efficient and diverse drug screening options and bringing better choices to clinical practice. Thus, we anticipate making clinically significant progress in the selection of second-line drugs after establishing 3D bioprinting models for GIST, aiming to identify effective treatment plans for patients that reduce treatment costs and alleviate adverse reactions. We currently report as follows:

## 1 Materials and methods

### 1.1 Establishment of patient inclusion criteria and primary cell isolation and culture

(1) This study included GIST patients who progressed after first-line treatment with tumor reduction surgery or localization puncture (percutaneous or ultrasound-guided endoscopic puncture) at our hospital. Inclusion criteria: 1) GIST patients who progressed after first-line treatment with tumor reduction surgery or localization puncture; 2) age ≥ 18 years, regardless of gender; 3) surgical resection or localization puncture to obtain at least one tumor sample with a volume greater than 0.5 cm^3^; 4) good general condition, expected to receive subsequent adjuvant therapy; 5) voluntarily signed informed consent. Exclusion criteria: 1) patients with poor general condition, expected to be intolerant to systemic treatment; 2) unable to obtain tumor samples through tumor reduction surgery or localization puncture; 3) poor patient compliance; 4) history of other malignant tumors.
(2) Collect surgical tumor specimens from GIST patients who progressed after first-line treatment: 1) preoperative preparation: patients were evaluated through clinical examination, imaging studies (computed tomography or magnetic resonance imaging), and laboratory tests to confirm the diagnosis and assess the extent of the tumor; 2) Surgical procedure: tumor resection was performed by experienced surgeons. The surgical approach was chosen based on the location, size, and extent of the tumor. For patients with localized tumors, complete resection with negative margins was attempted. For patients with metastatic or recurrent tumors, debulking surgery was performed to obtain sufficient tumor tissue for analysis. 3) Tissue sampling: during surgery, multiple samples were collected from different parts of the tumor to ensure that heterogeneity was captured. Each sample was approximately 0.5 cm^3^ in volume. Tissue samples were immediately placed in sterile containers with cold transport medium (Dulbecco’s modified eagle medium [DMEM] supplemented with 10% fetal bovine serum (FBS)) and transported to the laboratory within 30 min. 4) Postoperative handling: tissue samples were processed within 1 h of arrival in the laboratory. The samples were washed with sterile PBS to remove excess blood and debris. Tumor tissue was minced into small pieces (1–2 mm^3^) using sterile scalpels. The minced tissue was then digested with a mixture of collagenase type I (1 mg/mL) and DNase I (0.1 mg/mL) in DMEM at 37°C for 1–2 h with gentle agitation.
(3) Cell isolation and culture: After digestion, the tissue suspension was passed through a 70 µm cell strainer to obtain a single-cell suspension. The cell suspension was centrifuged at 1,000 rpm for 5 min, and the pellet was resuspended in complete culture medium (DMEM supplemented with 10% FBS, 1% penicillin–streptomycin, and 1% l-glutamine). Cells were cultured in a humidified incubator at 37°C with 5% CO_2_. Cell viability was assessed using the trypan blue exclusion method, and only samples with viability >90% were used for further experiments.

**Informed consent:** Informed consent has been obtained from all individuals included in this study.
**Ethical approval:** The research related to human use has been complied with all the relevant national regulations and institutional policies and in accordance with the tenets of the Helsinki Declaration, and has been approved by the authors’ institutional review board or equivalent committee.

### 1.2 Establishment of patient-derived GIST 3DP models

After successfully isolating and counting primary tumor cells, they were directly resuspended in bio-ink (Bio-ink composition: Printing material: GelMA (GelMA30, EFL). Final cell concentration: 1.0 × 10⁷ cells/mL in 7.5% (w/v) GelMA30 (with 0.1% (w/v) lithium phenyl-2,4,6-trimethylbenzoylphosphinate as a photoinitiator. Printing parameters: Nozzle diameter: 23G needle, printing speed 4.8 mm/s, extrusion speed 1.05 mm^3^/s, crosslinking under 405 nm light of 15 mW/cm² for 18 seconds; model parameters: grid structure, 6 mm × 6 mm × 0.92 mm, 4 layers, layer height 0.23 mm, line distance 0.99 mm; printing temperature: nozzle 23°C, bed 10°C) for bioprinting and cultured in GIST 3DP culture medium. Cell viability was tested on days 1, 3, 7, 14, and 28 to confirm that primary tumor cells can survive long-term in the 3D printing culture system.

### 1.3 Confirmation of the tumor 3D printing model retaining histological characteristics of parent tumor

The GIST biomarkers in the GIST-3DP model were detected to confirm that the tumor 3DP model highly retains the histological characteristics of the parent tumor.

### 1.4 This study investigates the efficacy and safety of personalized treatment for GIST patients who progressed a fter first-line imatinib, comparing their survival outcomes with those of a historical cohort

This study plans to include 10 cases of GIST patients who progressed after first-line treatment with imatinib and from whom tumor specimens can be obtained, forming a personalized treatment group. Patients in the personalized treatment group will select treatment plans based on drug sensitivity test results, and the drugs included in the selection will all be within the indications for that tumor, with controllable treatment risks. A retrospective statistical analysis of the progression-free survival (PFS) and overall survival (OS) of 52 GIST patients who progressed after first-line treatment at our hospital from January 2020 to April 2024 will be conducted. The PFS of the personalized treatment group will be compared with that of corresponding patients with GIST who progressed after first-line treatment admitted to our hospital during the earlier phase of the study, and treatment safety will be assessed.

### 1.5 Analysis of the correlation between the targeted drug response of GIST-3DP/GIST-3DMP models and the clinical response to targeted therapy in corresponding patients after first-line treatment progression

(1) collection of patient clinical data: Collect clinical data from patients, including their clinical response to targeted therapy. (2) Correlation analysis: conduct a correlation analysis between the drug response results from the 3DP models and the clinical response to targeted therapy in corresponding patients after first-line treatment progression.

## 2 Result

**Figure 1:**
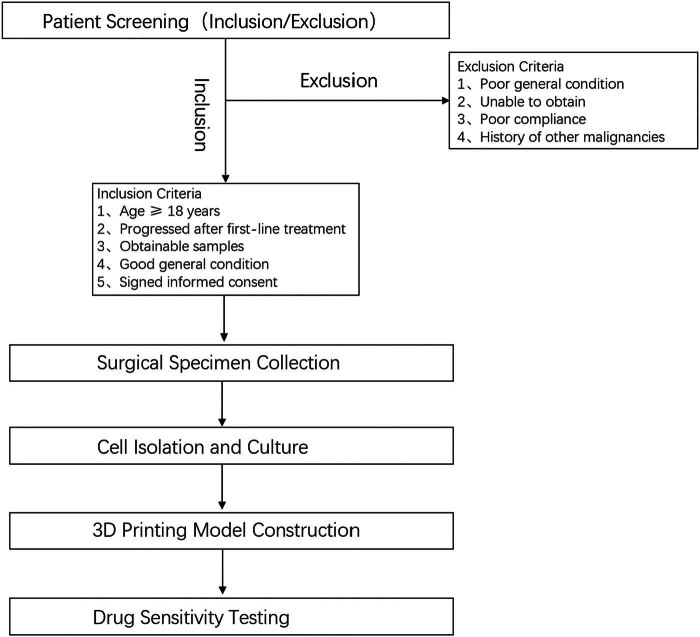
Flowchart of sample selection and processing.

**Figure 2:**
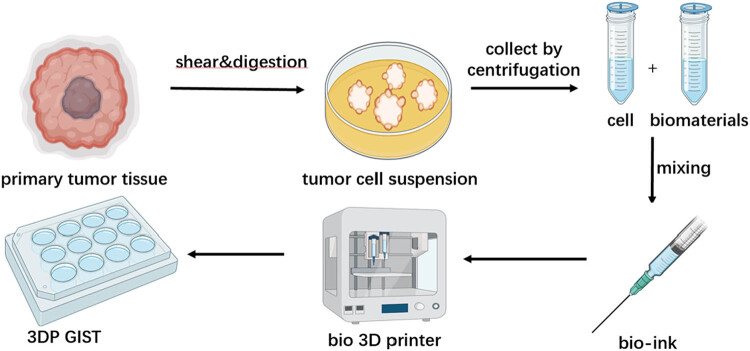
Flowchart of the establishment of 3DP tumor mode.

**Figure 3:**
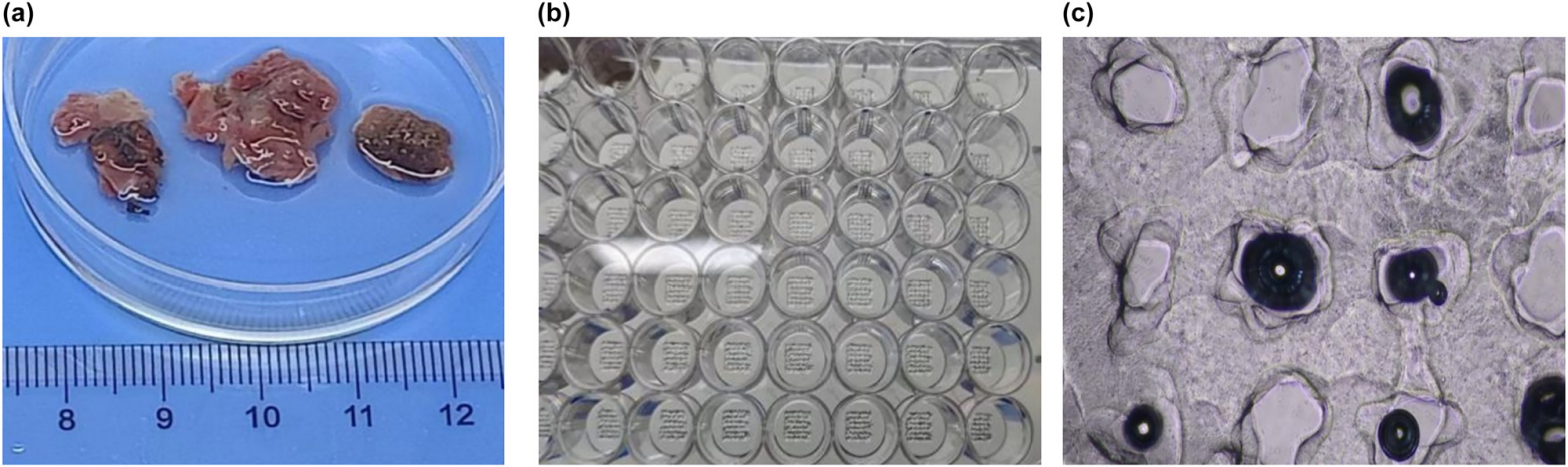
Fabrication of 3DP tumor models. Photographs of freshly resected tumor specimens (a), GIST 3DP models in 48-well plate (b), and bright field image of 3DP tumor models (c).

**Figure 4:**
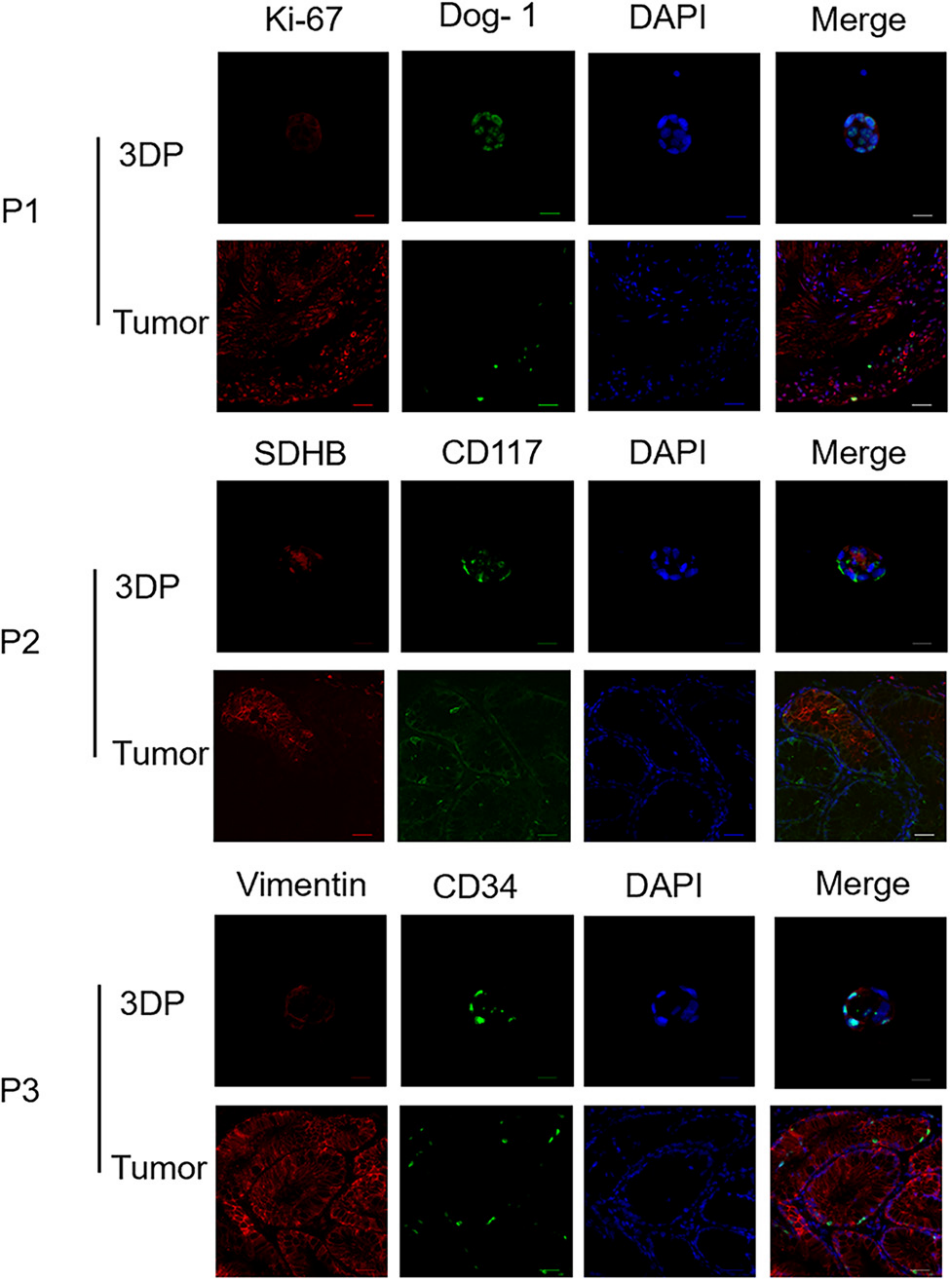
Immunofluorescence of 3DP tumor models and primary tumor tissues.

**Figure 5:**
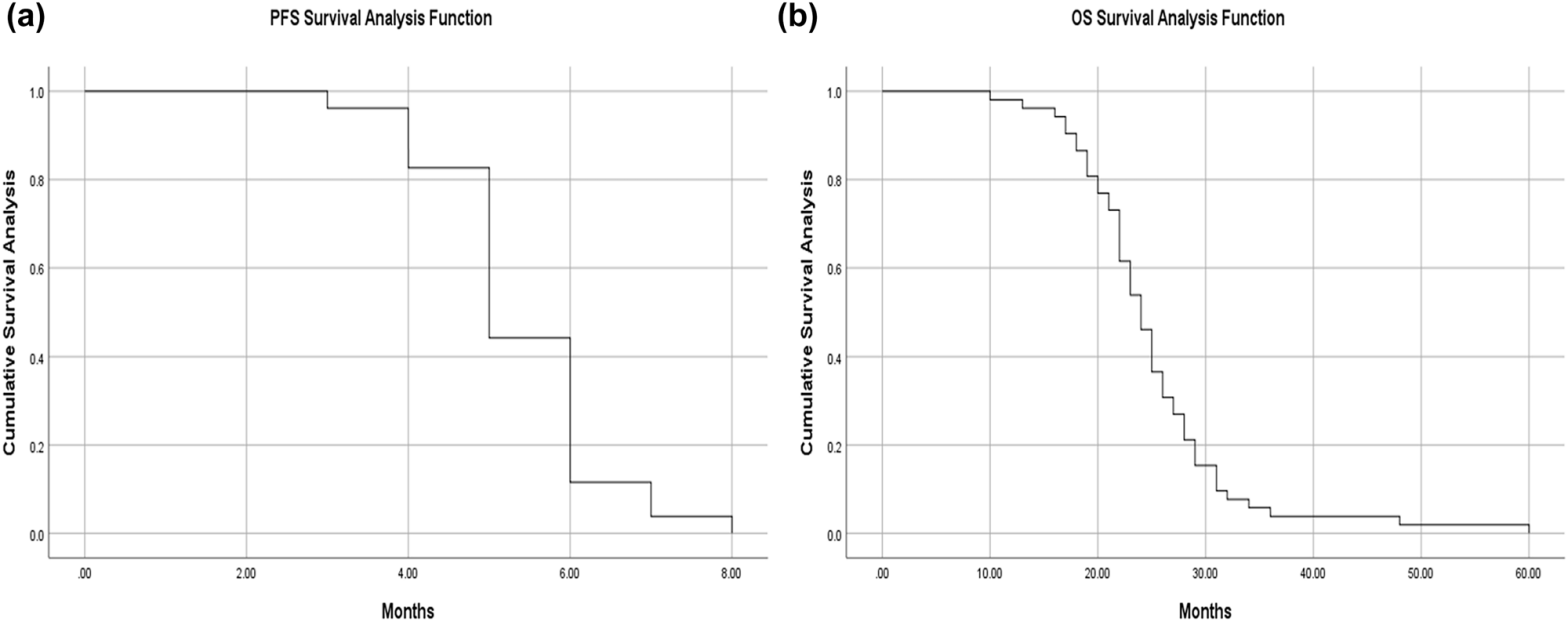
Survival analysis of PFS in patients with GIST in the pretreatment group (a) and survival analysis of OS in patients with GIST in the pretreatment group (b).

**Figure 6:**
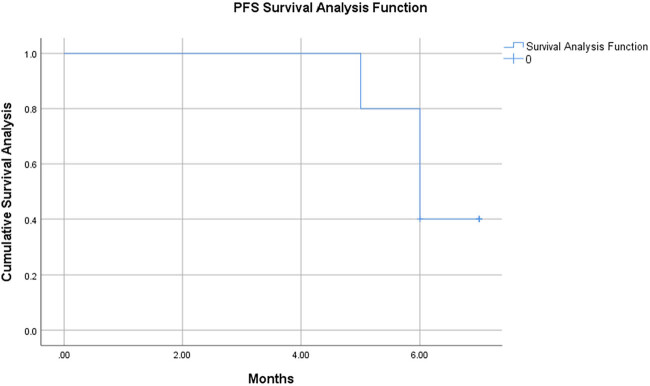
Survival analysis of PFS in patients with GIST in the individualized treatment group.

2․1 Flowchart illustrates the criteria for sample selection and the process of surgical specimen collection, cell isolation, 3D printing model construction, and drug sensitivity testing (Figure 1).
2․2 We established the process of creating patient-derived 3DP tumor models (Figure 2).
2․3 We collected surgical specimens, fabricated 3DP tumor models and photographs of freshly resected tumor specimens (Figure 3a), and have successfully been used to construct GIST 3DP tumor models: GIST 3DP models in 48-well plate (Figure 3b); bright field image of 3DP tumor models (Figure 3c).
2․4 We used immunofluorescence staining technology to observe the tumor markers in the 3DP tumor models and their corresponding parent tumor tissues separately. We found a high degree of consistency between the two, indicating that the 3DP tumor models highly retain the histological characteristics of the parent tumor tissues (Figure 4).
2․5 We retrospectively analyzed and calculated the PFS and OS of GIST patients who progressed after first-line treatment and were admitted to our hospital from January 2020 to April 2024 (a total of 52 cases), and performed survival analysis (Figure 5).
2․6 We included the clinical baseline information of 10 cases of GIST patients who progressed after first-line treatment (Table 1), performed PFS survival analysis (Figure 6), and compared the PFS between the previous treatment group and the personalized treatment group (Table 2) as follows:
2․7 The correlation analysis between the drug response of the 3DP tumor model and the clinical response to targeted therapy in patients is as follows:

**Table 1:** Clinical baseline information of GIST patients

Age	Gender	Tumor type	Gene testing	Drug sensitivity test results			
				Imatinib	Sunitinib	Regorafenib	Ripretinib
42	Man	Intestinal stromal tumor with liver metastasis	KIT p.K558_D572del mutation	Insensitive	Intermediate	Intermediate	Sensitive
47	Man	Recurrent gastric stromal tumor after surgery	KIT p.W557_K558del mutation	Intermediate	Intermediate	Intermediate	Sensitive
69	Female	Recurrent intestinal stromal tumor	KIT exon 9 non-synonymous insertion mutation	Insensitive	Sensitive	Sensitive	Insensitive
68	Man	Recurrent gastric stromal tumor after surgery	KIT p.k550_K557delinsiR mutation	Insensitive	Sensitive	Sensitive	Sensitive
62	Man	Recurrent gastric stromal tumor after surgery	KIT exon 11 mutation positive, exon 17 mutation positive	Insensitive	Intermediate	Sensitive	Insensitive
36	Female	Recurrent gastric stromal tumor after surgery	No mutations found in KIT and PDGFRA genes	Insensitive	Insensitive	Insensitive	Sensitive
61	Female	Recurrent gastric stromal tumor after surgery	KIT exon 11 small fragment insertion mutation	Insensitive	Insensitive	Intermediate	Insensitive
35	Man	Recurrent gastric stromal tumor after surgery	KIT gene exon 11 mutation	Insensitive	Insensitive	Intermediate	Intermediate
70	Man	Recurrent intestinal stromal tumor after surgery	KIT exon 11 mutation positive	Insensitive	Insensitive	Sensitive	Insensitive
54	Female	Recurrent gastric stromal tumor after surgery	KIT exon 9 mutation positive	Insensitive	Sensitive	Intermediate	Insensitive

**Table 2:** PFS of previous treatment group and personalized treatment group

Group	Total number of cases	Median PFS	*t*-value	*P*-value
Previous treatment group	52	5.3	−2.019	<0.05
Personalized treatment group	10	6.1		

Analysis of the clinical response to targeted therapy in a case of GIST patient admitted earlier is as follows: the patient had a recurrence of small intestine stromal tumor and underwent multiple surgeries. The genetic testing report showed that the primary gene mutation was a c-KIT exon 9 mutation. The treatment process was as follows: Gleevec prophylactic medication – increased dosage after progression – surgery after further progression – developed high fever after switching to Sutent – could not tolerate third-line treatment – progressed on fourth-line medication – received second-line treatment again (partial progression, partial effectiveness).

The clinical response to targeted therapy in the patient was highly consistent with the drug response of the 3DP tumor model. Moreover, the drug sensitivity test found that the single targeted drugs, imatinib and ripretinib, were not sensitive (consistent with the clinical presentation of this patient). However, when these two drugs were used in combination, they were found to be effective. This finding provides an explanation for the clinical phenomenon that the combination of targeted drugs is effective in some patients.

## 3 Discussion

Currently, two-dimensional (2D) culture is an important model for drug screening and has been widely applied in the medical field. Although it has the advantages of simplicity, reproducibility, and technical maturity, the planar 2D structure is significantly different from the three-dimensional (3D) spatial structure of the human or animal body. Antitumor drugs that show significant tumor-suppressing effects in 2D culture models may not have good pharmacological effects when applied to the human body [9]. In 2D cell line drug sensitivity tests, the fourth-line drug ripretinib has inhibitory effects on all secondary gene mutations except for the PDGFA D842V mutation. In actual clinical use, in head-to-head comparisons of drugs after first-line treatment progression, ripretinib did not show an advantage over sunitinib [10]. This to some extent also indicates the inconsistency between 2D cell line drug sensitivity tests and actual clinical situations. Therefore, its benefits to clinical practice are limited. Therefore, in recent years, the dynamics and complexity of the tumor microenvironment (TME) have been the main cause of this clinical dilemma. The TME consists of tumor cells, stromal cells, extracellular matrix, and various cytokines secreted by cells, which endow tumors with innate chemoresistance [11]. Studies have found that the TME plays an important role in tumor proliferation, invasion, metastasis, and the formation of drug resistance [12,13]. The individualized drug sensitivity detection platform based on bio-3D printing technology has extremely excellent precision, feasibility, and efficiency, giving this method an unbeatable advantage in establishing the required models, and it can efficiently and truly reflect the TME [14,15]. From past experience, tumor models constructed based on 3D bioprinting technology are structurally stable, have high success rates, low heterogeneity, and high consistency with tumors in the patient’s body. Combined with clinical practice, it shows that the results of drug sensitivity tests are highly reliable [16,17,18]. In particular, this detection platform can provide drug sensitivity test results in about 1 week, making it the only solution that can provide a test report before the clinical decision on the patient’s adjuvant treatment plan. The model construction success rate is close to 100%, which is significantly higher than other tumor models.

At present, in the realm of first-line treatment, the genotyping of GIST has successfully established a clear correlation with the application of targeted drugs. It can be said that for newly diagnosed GIST patients, individualized treatment based on tumor gene testing results is now possible. However, for patients who have progressed after first-line treatment, the specific medication and especially how to use medication based on gene testing results, although some progress has been made in clinical and research settings, it is still not clear. In particular, for the drug selection after first-line treatment progression in advanced GIST patients, the current guidelines suggest using drugs in a stepwise manner, and after fourth-line progression, it is recommended to participate in clinical trials or switch to previously effective drugs. A considerable number of experts have proposed that for GIST patients who have progressed after first-line treatment, the choice of second-line drugs should be based on gene testing results, but this view is still in question. Especially in the selection of second-line drugs, the controversy is more concentrated at present. Moreover, there are still many problems with the use of targeted drugs in the second-line clinical practice of GIST patients. First, in different historical periods or different centers, the acquisition of gene mutation data in advanced GIST patients is not consistent. The secondary mutations found in tissue samples and the secondary mutations found in blood circulating tumor DNA (ctDNA) are used as variables, and when comparing the efficacy of the third-line drug regorafenib, significant differences were found [19]. In addition, in advanced GISTs, there is significant heterogeneity between different recurrence foci, which also limits the clinical significance of local sample gene testing [20]. The positive results of blood circulating tumor DNA (ctDNA) mutation tests also show significant temporal heterogeneity with the use of sensitive targeted drugs [21].

Our study included 10 cases of GIST in the personalized treatment group and compared them with the 52 cases of GIST that progressed after first-line treatment and were admitted to our hospital in the past. It was found that the median progression-free survival (mPFS) of patients in the personalized treatment group was 6.1 months, which was higher than the mPFS of 5.3 months in the previous treatment group, and there was a statistical difference. Due to the insufficient follow-up time, it is not possible to compare the OS of patients. The findings of our study align with several other studies that have explored the potential of 3D bioprinting technology in the context of GIST treatment. For example, a study by Sun et al. [7] demonstrated that 3D-bioprinted colorectal cancer models effectively retained the histological and genetic characteristics of the parent tumors, showing a strong correlation between drug responses in the 3D models and clinical outcomes. This supports our observation that 3D-bioprinted GIST models can accurately reflect the TME and provide reliable drug sensitivity data. Moreover, the work by Bauer et al. [10] highlighted the limitations of traditional 2D cell line models in predicting clinical outcomes for GIST patients, particularly in the context of drug resistance. This further underscores the need for more advanced models like 3D bioprinting to better understand the complex interactions within the TME. Our study’s results, which show a higher PFS in the personalized treatment group using 3D bioprinting, provide additional evidence for the potential benefits of this technology. In addition, recent advancements in the understanding of GIST’s genetic landscape, as reported by several studies [19,20,21], highlight the challenges associated with genetic heterogeneity and the limitations of current gene testing methods. Our study addresses these challenges by using 3D bioprinting to create patient-specific models that can provide more accurate and personalized drug sensitivity data, potentially overcoming the limitations of traditional genetic testing.

## 4 Conclusion

Therefore, we have reason to believe that the 3DP model can be a powerful tool to participate in achieving personalized precision treatment as an *in vitro* tumor model that truly simulates the TME in the body, providing drug references for patients in a short time, selecting the optimal treatment plan, and strive to secure treatment time for patients, achieving the medical purpose of “surrogate drug testing.” We will include more cases and extend the patient follow-up time in the future to make the trial results more reliable. We look forward to making clinically significant progress in the selection of second-line drugs after the establishment of 3D bioprinting models for GIST.
